# Partial Synthetic PPARƳ Derivative Ameliorates Aorta Injury in Experimental Diabetic Rats Mediated by Activation of miR-126-5p Pi3k/AKT/PDK 1/mTOR Expression

**DOI:** 10.3390/ph15101175

**Published:** 2022-09-22

**Authors:** Yasmin M. Ahmed, Raha Orfali, Nada S. Abdelwahab, Hossam M. Hassan, Mostafa E. Rateb, Asmaa M. AboulMagd

**Affiliations:** 1 Department of Pharmacology and Toxicology, Faculty of Pharmacy, Nahda University, Beni-Suef 62521, Egypt; 2 Department of Pharmacognosy, College of Pharmacy, King Saud University, P.O. Box 2457, Riyadh 11451, Saudi Arabia; 3 Department of Pharmaceutical Chemistry, Faculty of Pharmacy, Nahda University, Beni-Suef 62521, Egypt; 4 Department of Pharmaceutical Analytical Chemistry, Faculty of Pharmacy, Beni-Suef University, Beni-Suef 62521, Egypt; 5 Department of Pharmacognosy, Faculty of Pharmacy, Beni-Suef University, Beni-Suef 62521, Egypt; 6 School of Computing, Engineering & Physical Sciences, University of the West of Scotland, Paisley PA1 2BE, UK

**Keywords:** type 2 diabetes mellitus (T2D), peroxisome proliferator-activated receptor (PPAR), intracellular adhesion molecule 1 (ICAM-1), endothelial nitric oxide synthase (eNOS), endothelin-1 (ET-1)

## Abstract

Type 2 diabetes mellitus (T2D) is a world wild health care issue marked by insulin resistance, a risk factor for the metabolic disorder that exaggerates endothelial dysfunction, increasing the risk of cardiovascular complications. Peroxisome proliferator-activated receptor PPAR) agonists have therapeutically mitigated hyperlipidemia and hyperglycemia in T2D patients. Therefore, we aimed to experimentally investigate the efficacy of newly designed synthetic PPARα/Ƴ partial agonists on a High-Fat Diet (HFD)/streptozotocin (STZ)-induced T2D. Female Wistar rats (200 ± 25 g body weight) were divided into four groups. The experimental groups were fed the HFD for three consecutive weeks before STZ injection (45 mg/kg/i.p) to induce T2D. Standard reference PPARƳ agonist pioglitazone and the partial synthetic PPARƳ (PIO; 20 mg/kg/BW, orally) were administered orally for 2 weeks after 72 h of STZ injection. The aorta tissue was isolated for biological ELISA, qRT-PCR, and Western blotting investigations for vascular inflammatory endothelial mediators endothelin-1 (ET-1), intracellular adhesion molecule 1 (ICAM-1), E-selectin, and anti-inflammatory vasoactive intestinal polypeptide (VIP), as well as microRNA126-5p and p-AKT/p-Pi3k/p-PDK-1/p-mTOR, endothelial Nitric Oxide Synthase (eNOS) immunohistochemical staining all are coupled with and histopathological examination. Our results revealed that HFD/STZ-induced T2D increased fasting blood glucose, ET-1, ICAM-1, E-selectin, and VIP levels, while decreasing the expression of both microRNA126-5p and p-AKT/p-Pi3k/p-PDK-1/p-mTOR phosphorylation. In contrast, the partial synthetic PPARƳ derivative evidenced a vascular alteration significantly more than reference PIO via decreasing (ET-1), ICAM-1, E-selectin, and VIP, along with increased expression of microRNA126-5p and p-AKT/p-Pi3k/p-PDK-1/p-mTOR. In conclusion, the partial synthetic PPARƳ derivative significantly affected HFD/STZ-induced T2D with vascular complications in the rat aorta.

## 1. Introduction

Type 2 diabetes (T2D) is a worldwide concern that establishes a significant influence on patient mortality and morbidity [1], affecting about 463 million adult people aged 20–79 years [2], which is expected to increase by 51% to 700 million by 2045 [2,3]. T2D is characterized by peripheral insulin resistance [4,5], which diminishes glucose reuptake in skeletal muscle and adipose tissue, leads to defective hepatic glucose output, and impairs insulin production from pancreatic B-cells [6,7]. Prolonged insulin resistance develops micro- and macrovascular problems predisposing to vascular risk factors, elevated blood pressure, obesity, diminished glucose metabolism, and dyslipidemia [8,9,10], consequently leading to microangiopathy in multiple organs, retina, kidney, and neurons, endothelial dysfunction, and risk of cardiovascular disease [11,12]. This metabolic syndrome increases free fatty acids; oxidative stress mediators induce the breakdown of mitochondrial functions [13,14] and impair endothelial nitric oxide synthase (eNOS) activity [15]. Elevated levels of endothelin-1 (ET-1) are produced as a result of decreased eNOS expression and increased vascular oxidative stress [16,17], as well as adhesion molecules resembling P-selectin and E-selectin [18,19].

Endothelial dysfunction is a hallmark of type 2 diabetes and a precursor to the development and worsening of atherosclerotic plaques [20], characterized by inflammation of the arterial wall controlled by vascular smooth muscle cells (VSMCs), macrophages, and endothelial cells (ECs) [21]. The lipotoxicity of saturated long-chain fatty acids in cardiomyocytes [22] is related to many factors such as reactive oxygen species (ROS) [23], peroxisome proliferator-activated receptors (PPARs) [24,25], and phosphoglycerate cofactor 1 (PGC-1) [26]. Furthermore, endothelial short noncoding microRNAs (miRNAs) have essential roles in vascular formation, hemodynamic stress, progression of atherosclerosis, and inflammation [27,28]. The most abundant meta-regulators for endothelial gene expression miRNAs are miR-126-3p and miR-126-5p [28]; the aberration for gene-encoding pre-miR-126 impacts vascular integrity and angiogenesis [29]. On the other hand, the significant presence of ischemic neovascularization in the *Mir126*^−/−^ mice model and the transmission of miR-126-3p via microparticles released from apoptotic ECs inhibits atherosclerosis [30,31,32], demonstrating that miR-126 is essential for the endothelium stress response. Furthermore, the endothelial cell death inhibitor miR-126-5p works by directly targeting the transient receptor potential channel (TRPC6) [28,33]. Tang et al. [34] revealed that the overexpression of miR-126-5p triggers the Phosphatidylinositol-3-Kinase/Serine-Threonine Kinase/Mammalian/Mechanistic Target of Rapamycin (PI3K/Akt/mTOR) pathway by restoring autophagy, reflecting the antiatherogenic effect of miR-126-5p [35]. Consequently, cell proliferation, migration, and survival of endothelium and VSMCs are all improved by activating PI3K/Akt/mTOR [36,37]. The upregulation of adenosine monophosphate-activated protein kinase–mammalian/mechanistic target of rapamycin (AMPK/mTOR) and hypoxia-inducible factor alpha (HIFα) is related to the induction of autophagy [38], while PI3K/Akt/PDK1/mTOR, peroxisome proliferator-activated receptors gamma (PPARƳ), and nuclear factor kappa B (NF-κB) are significant to reserve autophagy [39]. Dong et al. [40] recently revealed that miRNA-126-5P significantly interacted with peroxisome proliferator-activated receptor alpha (PPARα), ATP-binding cassette transporter (ABCA1), and cholesterol 7α-hydroxylase (CYP7A1) genes, ameliorating dyslipidemia and atherosclerosis.

The nuclear receptor superfamily includes ligand-activated transcription factors called peroxisome proliferator-activated receptors (PPARs) [28]; the PPAR family is subdivided into three isotypes PPARα, PPAR β/δ, and PPARƳ [41]. Activation of the PPARs subtype is essential for controlling cell proliferation, differentiation, apoptosis [42], enhancing cell development [43], and wound healing [44], raising high-density lipoprotein (HDL) levels [45], reducing triglyceride levels [46], and improving insulin sensitivity [47]. However, PPARα is widely distributed throughout the body tissues such as cardiac [48], renal, liver [49], muscles, and adipose tissue [50], which is essential for regulation of angiogenesis, inflammation, and free fatty acid catabolism [51]. Recent studies indicate that the ECs, VSMCs, and macrophages co-expressed both isotypes PPARα and PPARƳ [52], which function in endothelial cell survival and proliferation [53]. It has been reported in individuals with T2D exposed to high-fat meals that protein, lipid, and carbohydrate load was connected to increased ROS generation and impaired endothelium-dependent vasodilation [54], lowering the endothelial function [55,56]. The activation of vascular endothelial cells results in the release of pro-inflammatory adhesion molecules such as Intracellular Adhesion Molecule 1 (ICAM-1), Vascular Cell Adhesion Protein 1 (VCAM-1), and E-selectin expression [57], as well as an increase in pro-inflammatory cytokines such as tumor necrosis factor-, interleukins, and platelet-derived growth factor [58]. The simultaneous activation of dual alpha and gamma PPARs agonists may provide superior glucose and lipid regulation compared to single subtype-selective drugs [59,60]. In addition, growth factors and cytokines that promote endothelial cell migration also regulate angiogenesis [61,62], proliferation [63], and survival to promote revascularization and tissue ischemia affected by T2D [64,65]. 

Currently, thiazolidinedione (TZDs), such as pioglitazone, ciglitazone, troglitazone, and rosiglitazone and their composites are essential drugs promoting favorable effects in modulating endothelial dysfunction in T2D comorbidity due to their anti-inflammatory and anticancer effects, as well as antihyperlipidemic activity [66,67]. They act on PPARα and PPARƳ to ameliorate hyperlipidemia and hyperglycemia in T2D patients [68]. Consequently, they may downregulate the activation of proinflammatory mediators via Pi3k, AKT, and mTOR signaling pathways [69,70] by promoting favorable effects in modulating endothelial dysfunction in diabetes comorbidity due to their anti-inflammatory and anticancer effects, as well as antihyperlipidemic activity [71,72]. Additionally, PPARs are expressed in adipose tissue and endothelial cell lining [73], modulating chemokines and adhesion molecules (ICAM, VCAM), as well as downregulating ROS [74]. Indeed, PPARƳ enhances nitric oxide (NO) production in the endothelium and retracts ET-1 expression, promoting endothelial relaxation [75,76]. Ahmet et al. [77] reported that pioglitazone analogue significantly regulates Streptozotocin-Induced T2D through stimulating local angiotensin-converting enzyme 2/angiotensin 1-7 axis with the aid of PI3K/AKT/mTOR Signaling pathway in the hepatic tissues, thereby regulating glycogen deposition and enhancing lipolysis. However, Molavi et al. [78] found that PPARƳ ligand rosiglitazone protects against myocardial ischemia/reperfusion injury via an effect on AT2 receptor upregulation and p42/44 MAPK inhibition. Thus, the greater abundance of PPARs in different body organs may be promising to protect T2D patients from cardiovascular comorbidity.

Even though many previous studies demonstrated the beneficial effect of PPAR ligands in the treatment of T2D patients with cardiovascular complications and endothelial damage, to date, few studies have examined the beneficial effect of PPARƳ ligand agents on miR-126-5p and Pi3k/AKT/PDK1/mTOR expression in T2D-induced vascular damage. Our study designed a new partial synthetic PPARƳ ligand derivative to assess its protective effect on tissue-induced vascular changes in the aorta of diabetic rats; the aorta vascular tissue levels were estimated for ET-1, ICAM-1, E-selectin, and VIP, qRT-PCR microRNA126-5p gene expression and Western blotting expression of p-AKT/p-Pi3k/p-PDK-1/p-mTOR, coupled with immunohistochemical examination for endothelial nitric oxide synthase (eNOS) and histopathological examination using hematoxylin and eosin. Our findings revealed that synthetic derivatives upregulate miR-126-5p, enhancing p-Pi3k, p-AKT, p-PDK, and p-mTOR signaling pathway activation coupled with suppressing proinflammatory molecules ET-1, ICAM-1, E-selectin, and the anti-inflammatory vasoactive intestinal polypeptide (VIP) in diabetic rats. Furthermore, immunohistochemical estimations of eNOS and histopathological examination using hematoxylin and eosin for aortic tissues enhanced the role of partial synthetic PPARƳ derivatives in correcting diabetes-induced vascular complications.

## 2. Results

### 2.1. Effect of Streptozotocin on Serum Fasting Blood Glucose Adult Female Albino Rats with Experimentally-Induced Diabetes Mellitus 

The mean values of the normal control group regarding serum fasting blood glucose (mg/dL) were 101.66 ± 4.73. Rats subjected to STZ showed significantly higher fasting blood glucose serum levels, reaching 289.33 ± 12.90 (284.60% increase) compared to normal control rats. However, rats subjected to STZ + PIO as a standard treatment and STZ + P-PPARƳ synthetic derivative groups showed significantly decreased mean values of fasting blood glucose levels (129.66 ± 8.08 and 96.33 ± 0.8.14, respectively), respectively, when compared to those in the STZ group (Table 1).

### 2.2. Effect of 2 Weeks of Treatment with P-PPARƳ Synthetic Derivative on Tissue E-Selectin and ICAM-1 Level in Adult Female Albino Rats with Experimentally Induced T2D Vascular Damage 

**Figure 1:**
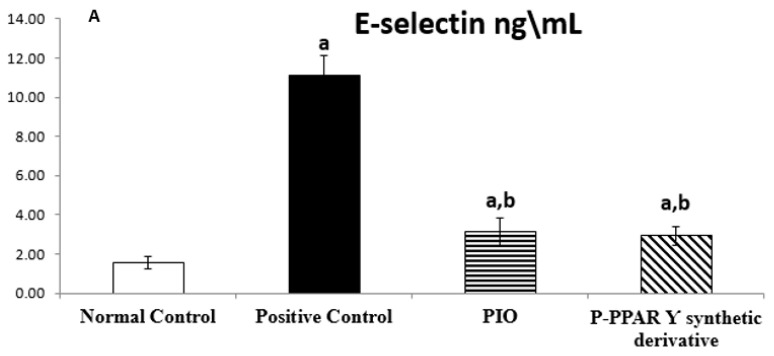

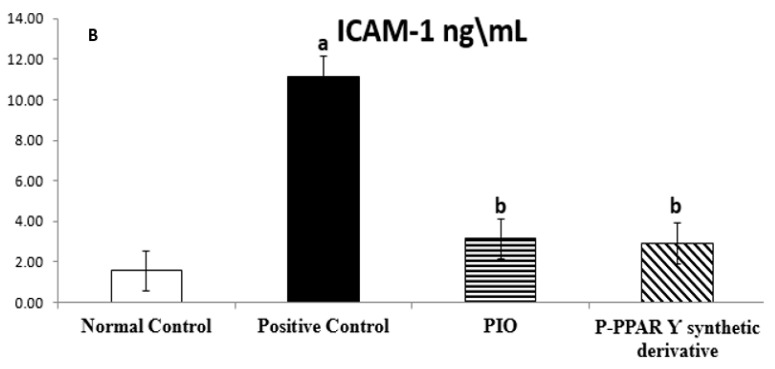
Bar chart illustrating 14 days of oral standard PIO and P-PPARƳ synthetic derivative treatment on aorta tissue levels of E-selectin (**A**) and ICAM-1 (**B**) against STZ-induced vascular damage. The data are shown as mean values with standard errors (SEM) at *p* < 0.05; ^a^ significantly different from the normal control group; ^b^ significantly differ from the positive control group.

The mean values of the normal control group regarding tissue protein intracellular adhesion molecule 1 (ICAM-1) (ng/mL) and E-selectin (pg/mL) were 14.50 ± 0.53 and 1.55 ± 0.30, respectively. The T2D group significantly increased ICAM-1 and E-selectin in tissue (688.69% and 718.71% increases, respectively), compared with normal rats. On the other hand, the standard PIO group represented an improvement in ICAM-1 level by 21.23% and E-selectin by 27.83% regarding the STZ positive control group. While the rats received P-PPAR γ synthetic derivative treatment significantly improved tissue ICAM-1 level to 28.70% and E-selectin to 26.30 compared with diabetic comorbidity rats. Treatment with P-PPAR γ synthetic derivative improved ICAM-1 and E-selectin substantially better than the reference standard PIO (Figure 1A,B).

### 2.3. Effect of 2 Weeks of Treatment with P-PPA Ƴ Synthetic Derivative on Tissue VIP and ET-1 Level in Adult Female Albino Rats with Experimentally Induced T2D Vascular Damage 

**Figure 2:**
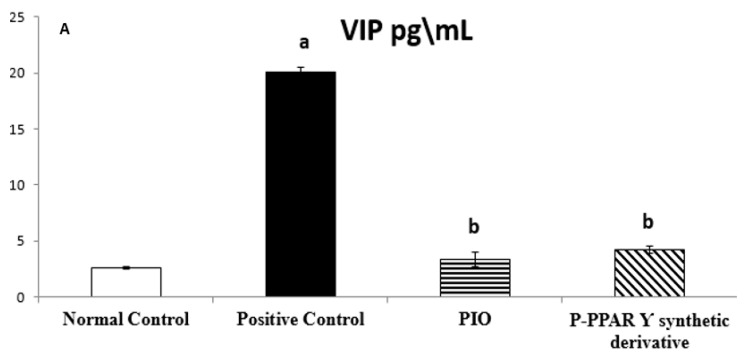

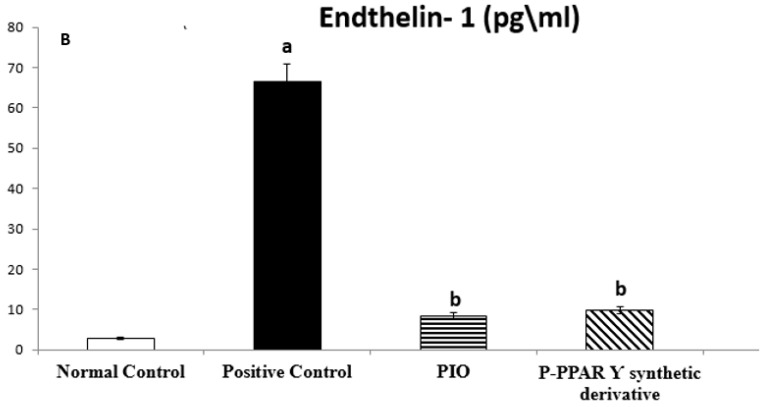
Bar chart illustrating 14 days of oral standard PIO and P-PPARƳ synthetic derivative treatment on aorta tissue levels of VIP (**A**) and ET-1 (**B**) against STZ-induced vascular damage. The data are shown as mean values with standard errors (SEM) at *p* < 0.05; ^a^ significantly different from the normal control group; ^b^ significantly differ from positive control group.

The mean values of normal control group regarding tissue VIP (pg/mL) and ET-1 (pg/mL) were 2.63 ± 0.11 and 2.88 ± 0.26, respectively. Rats subjected to the STZ positive control group exposed to a significant increase in the tissue levels of ET-1 and P-selectin, increasing by 767.30% and 2310.42%, respectively. However, the PIO standard group improved tissue levels of VIP by 16.60% and ET-1 by 12.73% compared to the STZ group, while the P-PPARƳ synthetic derivative revealed a significant improvement in VIP by 20.71% and ET-1 by 14.74% compared to STZ group that showed a better improvement of the derivative when compared to the reference standard PIO group (Figure 2A,B).

### 2.4. Effect of 2 Weeks of Treatment with P-PPARƳ Synthetic Derivative on Regulating miR-126-5p Gene Expression

**Figure 3:**
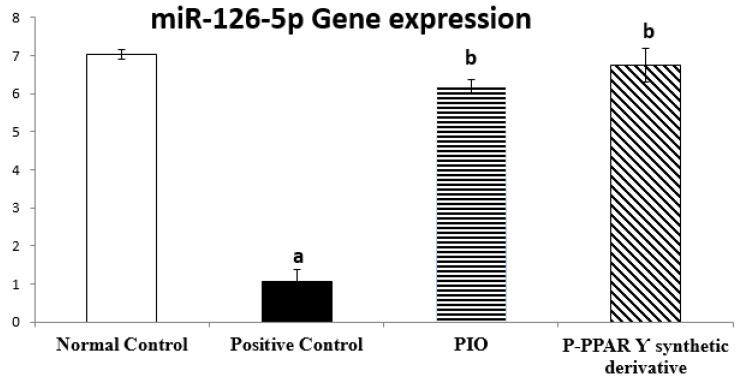
Bar chart illustrating 14 days of oral standard PIO and P-PPARƳ synthetic derivative treatment on aorta miR-126-5p against STZ-induced vascular damage. The data are shown as mean values with standard errors (SEM) at *p* < 0.05; ^a^ significantly different from the normal control group; ^b^ significantly differ from positive control group.

To determine miR-126-5p contribution in vascular repair induced by P-PPARƳ derivatives treatment against T2D in experimental rats, we used qRT-PCR to evaluate miR-126-5p gene expression. Notably, unlike the normal control rats group, diabetic rats significantly decreased miR-126-5p expression to 14.99%, compared to the normal control group. Oral treatment with pioglitazone and P-PPARƳ synthetic derivative (20 mg/kg, p.o) significantly upregulated miR-126-5p expression to 588.79% and 641.50%, respectively, compared to the diabetic positive control group. However, P-PPARƳ synthetic derivative treatments restored miR-126-5p gene expression back to normal. These results indicate that P-PPARƳ synthetic derivatives counteracted STZ-induced apoptosis and endothelial damage suggesting a functional involvement in regulating miR-126-5p expression-induced vascular endothelial repair (Figure 3).

### 2.5. Effect of 2 Weeks of Treatment with P-PPARƳ Synthetic Derivative on Activation of p-AKT/p-Pi3k/p-PDK 1/p-mTOR Expression, Enhancing Vascular Endothelial Repair 

**Figure 4:**
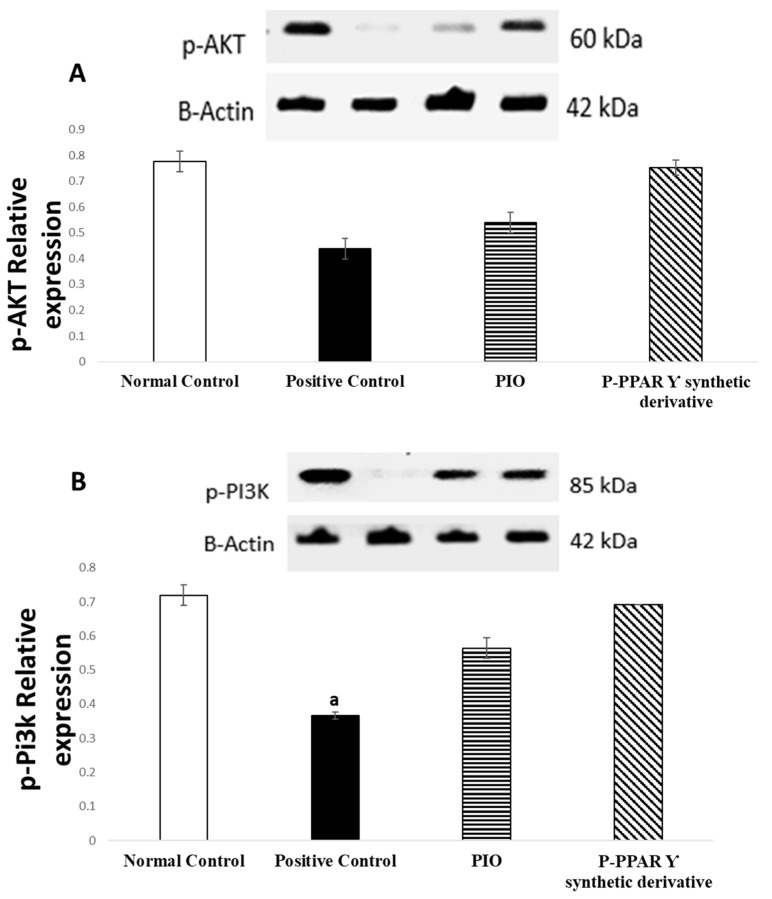

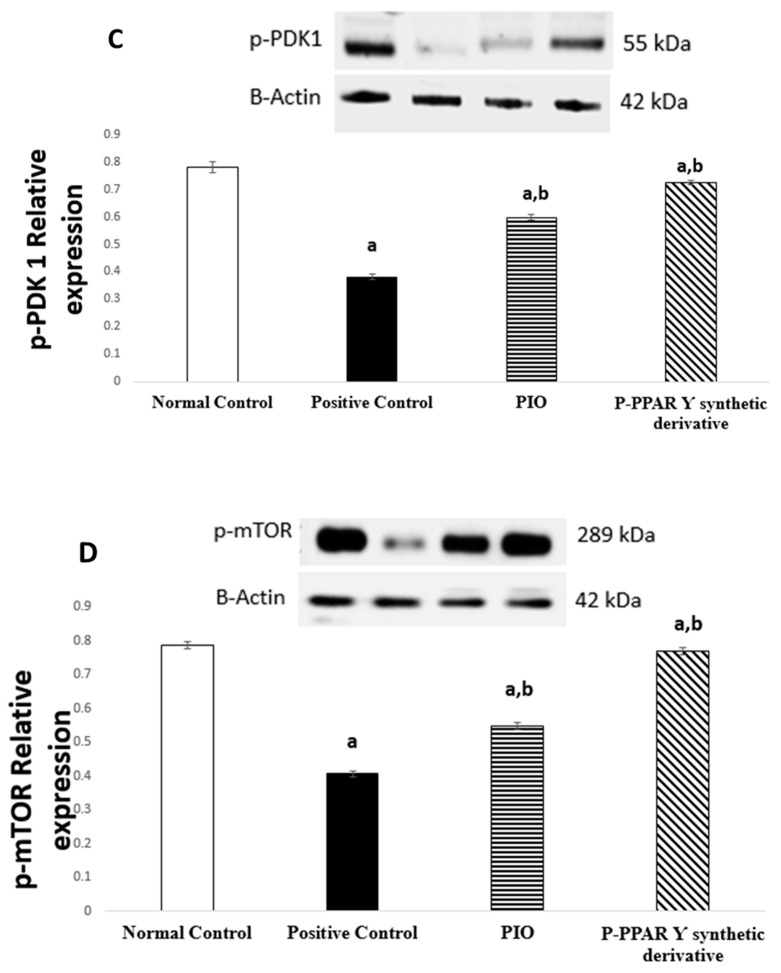
The bar chart and bands reflect the protein expression of aorta tissue toward (**A**) p-AKT, (**B**) p-Pi3k, (**C**) p-PDK, and (**D**) p-mTOR after 2 weeks of oral treatment with oral standard PIO and P-PPARƳ synthetic derivative treatment against STZ-induced vascular damage. The data are shown as mean values with standard errors (SEM) at *p* < 0.05; ^a^ significantly different from the normal control group; ^b^ significantly differ from positive control group.

Restoring the phosphorylation of the p-AKT/p-Pi3k/p-PDK/p-mTOR signaling pathways triggers the endothelium defense mechanism. Western blot analysis showed a significantly diminished expression of p-AKT/p-Pi3k/p-PDK 1/p-mTOR to 56.41%, 51.39%, 61.54%, and 43.82%, respectively, in diabetic rats than in normal control animals Alternatively, the treatment of rats with the standard PIO represented an improvement in p-AKT/p-Pi3k/p-PDK 1/p-mTOR expression, increasing by 162.16%, 154.55%, 145.83%, and 176.92%; additionally, rats receiving P-PPARƳ synthetic derivative significantly increased the expression of p-AKT/p-Pi3k/p-PDK 1/p-mTOR signaling pathways by 115.90%, 135.13%, 124.68%, and 138.56%, respectively, compared to the positive control group. Our results indicate that, with P-PPARƳ synthetic derivative treatment, p-AKT/p-Pi3k/p-PDK 1/p-mTOR signaling pathway expression was restored to normal levels (Figure 4A–D).

### 2.6. Effect of 2 Weeks of Treatment with P-PPARƳ Synthetic Derivative on Attenuating Histopathological Aortic Strip Endothelial Abrasions

**Figure 5:**
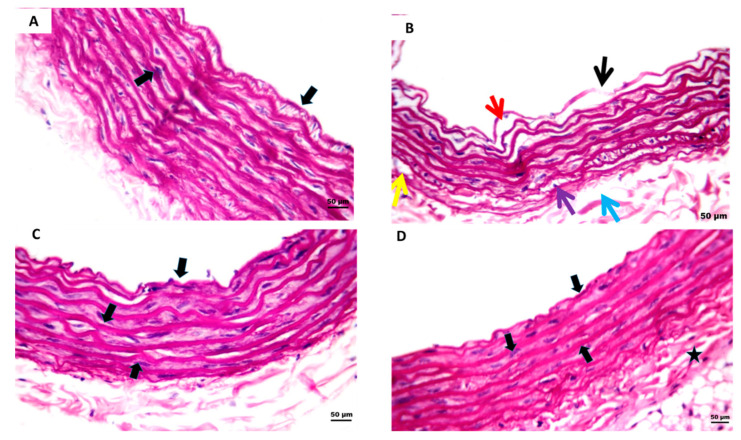
STZ-induced endothelial damage in rat aorta H&E staining sections. (**A**) The normal control presents elastic tissues with normal endothelium and smooth muscle cells, all represented by black arrows. (**B**) Positive control group showing intimal destruction (black arrow), with clefts in sub-intima (red arrow), media (yellow arrow) with cytoplasmic clearing of smooth muscle cells (violet arrow), and sub-medial separation (blue arrow), (**C**) Oral treated standard group PIO signifies minimal endothelial layer clefting, in addition to restoring normal smooth muscles, all represented by black arrows. (**D**) P-PPARƳ synthetic derivative compound showing integral intima (black arrow) with slight medial clefting (star) and normal smooth muscle cells (middle black arrows).

Histopathological examination was indicated to detect STZ-induced aortic endothelial blood vessel abrasions and the ability of P-PPARƳ synthetic derivatives to modulate endothelial texture against injury in comparison with standard group PIO. The aorta strip section revealed a normal endothelium and smooth muscle, regarding the normal control group. Additionally, it showed elongated nuclei with an eosinophilic cytoplasm-enhanced marked elastic tissue (Figure 5A). By contrast, the STZ positive control group showed average endothelial lining coupled with marked clefts in the media with cytoplasmic vacuoles in smooth muscle cells and sub-medial separation (Figure 5B). PIO standard treatment group showed minimal endothelial layer clefting (Figure 5C). In contrast, the P-PPARƳ synthetic derivative group decreased vascular endothelial pathological changes by returning endothelial blood vessels to their normal form with a slight smooth muscle clefting and restoring elastic tissue activity (Figure 5D).

### 2.7. Effect of 2 Weeks of Treatment with P-PPARƳ Synthetic Derivative on Mitigating ROS and Enhancing Tissue Antioxidant Defense Mechanism

**Figure 6:**
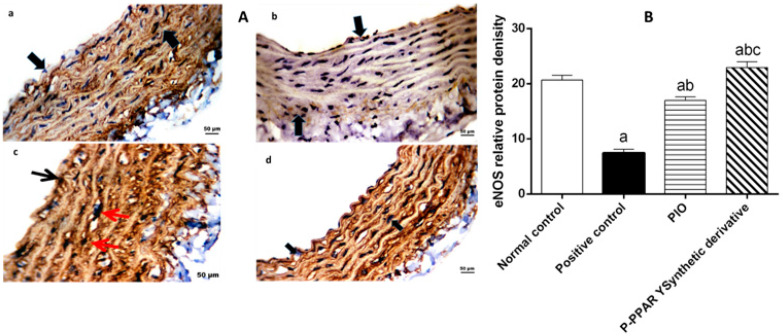
(**A**) A representative immunohistochemical examination of endothelial aorta rat sections with eNOS against STZ-induced damage followed by 2 weeks of treatment with oral standard PIO group and P-PPARƳ synthetic derivative. (**a**) Normal control section showing blood artery wall endothelium and smooth muscle cells exhibiting robust cytoplasmic responses to endothelial eNOS, all represented by black arrows. (**b**) Positive control group STZ representing the vascular aortic walls with weak eNOS reactivity in the cytoplasm of endothelial cells and smooth muscle cells, represented by black arrows. (**c**) PIO group representing mild cytoplasmic reactivity to eNOS in endothelial cells (black arrow) and in smooth muscle cells (red arrows); (**d**) P-PPARƳ synthetic derivative group highly expressed in the cytoplasmic responsiveness to eNOS and in the blood vessel wall represented by black arrows; (**B**) Graphical presentation of the changes in eNOS immunostaining intensity in different groups. Bars represent the mean ± SD (*n* = 4). Comparisons were made using one-way ANOVA followed by Tukey’s post hoc test. ^a^ Significantly different from control group at *p* < 0.05; ^b^ significantly different from positive control group; ^c^ significantly different from PIO group at *p* < 0.05.

An endothelial nitric oxide synthase (eNOS) expression assay investigated endothelial oxidative stress following STZ-induced vascular endothelial injury. Our data revealed that rats subjected to STZ showed a weak eNOS reaction in the endothelial lining and smooth muscle cytoplasm (Figure 6(Ab)) compared to the normal control group (Figure 6(Aa)). PIO standard treatment group endothelial cells showed a mild cytoplasmic reactivity to eNOS, with no reactivity on smooth muscles (Figure 6(Ac)), while P-PPARƳ synthetic derivative re-established eNOS expression on the cytoplasm and endothelial smooth muscles (Figure 6(Ad)) compared to the positive control group. The immunohistochemical findings reveal that eNOS expression increased after treatment with P-PPAR Ƴ synthetic derivative reaching normal control levels, demonstrating the role of tested drugs as antioxidants and ROS scavengers in modifying blood vessel activity (Figure 6A,B).

## 3. Discussion

Diabetes mellitus forms progressive diseases of the blood vessels and cardiomyopathy [79], as well as increases the rate of cardiac hypertrophy [5]. DM is characterized by the presence of elevated oxidative stress [80], elevated inflammatory and vascular biomarkers [81], disturbance in lipid metabolism [82], fibrosis, and elevated serum cardiac injury muscle biomarkers [83,84]. In the current study, we investigated the role of pioglitazone and a new naturally inspired P-PPARƳ synthetic derivative that improves endothelial enhancement through alleviating the inflammatory cascade and vascular endothelial modulation via upregulating endothelial miR-126-5p gene expression.

The STZ model is a well-established method for inducing type 1 or 2 diabetes in rats and, subsequently, diabetic complications [85]. For this purpose, our results represent that STZ aortic tissue levels significantly elevated VIP, E-selectin, endothelin-1, and ICAM-1 levels compared to the normal control group (Figure 1A). Moreover, severe endothelium smooth muscle histological abnormalities were evidenced by endothelial lining clefts with cytoplasmic vacuoles in smooth muscle proliferation, as well as eNOS in the smooth muscle (Figure 5A,B and Figure 6A,B).In agreement with our data results, previous studies revealed that STZ-induced DM endothelial complications in the experimental rats significantly increased tissue levels of VIP, E-selectin, endothelin-1, and ICAM-1 [86,87]. Additionally, earlier data proved that vascular damage is induced as a secondary complication to metabolic syndrome-induced insulin resistance in diabetic patients [88], enhancing immunological disorders [89,90]. Consequently, endothelial damage triggers systemic inflammation by increasing the production of proinflammatory molecules and vasoconstrictor agents such as VIP, E-selectin, endothelin-1, and ICAM-1 [91,92], together with an imbalance between endothelial eNOS and iNOS [93,94]. These results agree with our data that DM induction via different mechanisms mediates STZ action. 

Moreover, numerous studies have shown that hyperglycemia causes severe inflammation [86,87]. Researchers have outlined thiazolidinediones, especially pioglitazone and rosiglitazone, to manage endothelial problems [95]. Treatment with a PPARƳ agonist inhibits LPS-induced endothelial inflammation by reducing IL-6, VCAM, TNF-α, and mRNA expression [96]. In turn, this reduces the production of inflammatory mediators, adhesion molecules, and atherosclerosis in endothelial cells [97,98]. Similarly, a model of diabetic nephropathy suggested that treatment with pioglitazone reduces glomerular sclerosis, fibrosis, and hypertrophy by lowering ICAM-1, E-selectin, and albuminuria [98,99]. However, a study on women with polycystic ovarian syndrome found that pioglitazone treatment for insulin resistance dramatically improved endothelial-independent function, adipokines, and ET-1 [100]. Furthermore, VIP modulation played a significant role in carbohydrate and lipid metabolism [101], in addition to being a potent anti-inflammatory and neuroendocrine vasodilator [102]. Consequently, in an Alzheimer’s disease animal model, a new action on glial cell polypeptide was revealed, which shielded neurons against toxins and memory loss coupled with inhibiting oxidative stress production in the vascular compartment [103]. 

Additionally, in an STZ-induced diabetes mellitus rat model, pioglitazone administration for four weeks in a row restored ET-1, superoxide dehydrogenase (SOD), and NAD(P)H oxidase activity, thereby restoring aortic function [100,103]. Pitocco et al. [104] reported pioglitazone’s effectiveness in treating pulmonary hypertension rats via inhibiting cellular remodeling, proliferation, and inflammation of VSMCs. Correspondingly, the efficacy of thiazolidinediones in treating human endothelium by decreased inflammatory, pro-inflammatory, and vasoconstrictor agents has been reported [105]. Our findings are consistent with the findings of the prior study. These studies validated our results and confirmed the anti-inflammatory and anti-oxidant properties of the investigated agents.

Atherosclerosis-induced hypercholesterolemia is a cardiovascular progression coupled with type 2 diabetes mellitus [106], which increases reactive oxygen species (ROS), and subsequent eNOS degradation, releasing endothelin 1-induced vasoconstriction [107]. Changes in cholesterol levels due to glucose intolerance induce dysregulation of ICAM-1 and VCAM-1 [108]. miR-126-5p downregulation has been reported in elevated serum levels of ICAM-1, VCAM-1, and E-selectin-induced coronary syndrome [109]. Recent studies have revealed the critical role PI3K/AKT/mTOR axis in governing cell survival [82]. Additionally, Jia et al. [21] reported the crosstalk of the circRNA/PI3K/AKT axis, particularly regarding its protective effect against atherosclerosis, oxidative stress, and apoptosis via the regulating impact of tumor cell biological activities. In particular, miR-126-5p maintains a key function in the integrity of endothelial cells, inflammation, angiogenesis, and vascular repair [110]. Otherwise, recent data represent that miR-126 overexpression significantly increases the protein expression of the PI3K, Akt, GSK3β, and ERK1/2 signaling pathways and attenuates ROS vascular content [111]. Another research project reported that miR-126 negatively regulates vascular endothelial growth factor expression in hypoxia-induced monkey chorioretinal vessel endothelial cells [112]. 

Furthermore, it was discovered that pioglitazone plays a key role in reducing ventricular hypertrophy via ERK activation and increased phosphorylation of the AMPK axis in an experimentally induced hypertensive rat model [113]. It was recently reported that the STZ/HFD-induced insulin resistance [114] significantly suppresses p-AMPK, p-Pi3k, p-AKT, p-PDK, and p-mTOR axis levels in HepG2 cells [115], demonstrating that miRNAs play a role in heart illness as fundamental regulators of gene expression [116]. Additionally, pioglitazone targets miR-126-5p gene expression, which is involved in inflammatory processes, adhesion molecules, cell-cycle events such as proliferation and migration, apoptosis, and NO signaling in endothelial cell health, leading to the development of atherosclerosis [117,118,119]. Our study results revealed that treating diabetic rats with pioglitazone and P-PPARƳ synthetic derivatives associated with the significant upregulation of miR-126-5p expression (Figure 3) activated the expression and phosphorylation of the p-Pi3k, p-AKT, p-PDK 1, and p-mTOR axis (Figure 4), coupled with histopathological endothelial lining healing (Figure 5) and eNOS restoration in the endothelium (Figure 6).

## 4. Materials and Methods

### 4.1. Chemicals, Reagent Kits, Antibodies, and Tested Agents

Streptozotocin (Catalog number MFCD00006607) and pioglitazone hydrochloride (Catalog number MFCD04975446) were purchased from Sigma–Aldrich Chemical Company (St. Louis, MO, USA). Enzyme-linked immunosorbent assay (ELISA) kits for rat ET-1 (catalog number MBS5704215), E-selectin (Catalog number ERA14RB), ICAM-1 (Catalog number RAB0221-1KT), and eNOS (Catalog number PA5-17917) vasoactive intestinal peptide (VIP; catalog number MBS5031002) were obtained from ThermoFisher Scientific (Rockford, IL, USA). The Western blotting assay had different primer antibodies against P-pi3k (Catalog number sc-293115), P-AKT (Catalog number # 200-301-268), p-PDK-1 (Catalog number sc-515944), and P-mTOR (Catalog number # PA1-518). The qRT-PCR monoclonal antibody for miR-126-5p (Catalog number 217004) was obtained from Qiagen (Germantown, MD, USA). All materials were obtained from authorized sources in analytical grade.

### 4.2. Animals

The Nahda University animal house in Beni-Suef, Egypt, provided adult female albino rats weighing 200–220 g. Before starting the experiment, the animals were held in controlled conditions for 12/12 h with food and water access at the optimal temperature and humidity conditions. The treatment and care of the animals were conducted following the National Institutes of Health (NIH) Guide for the Care and Use of Laboratory Animals (Publication No. 85-23, revised 1985).

### 4.3. Animal Experimental Model

Adult female forty albino rats were divided into four groups:

Normal control group: receiving vehicle in Tween-80, 2%;

STZ (T2D) positive control group: receiving intraperitoneal STZ injection (45 mg/kg) [120] after being subjected to three consecutive weeks of HFD feeding [77];

Pioglitazone-treated group: receiving STZ + HFD as with positive control group, as well as pioglitazone (20 mg/kg/14 day, p.o) dissolved in Tween-80, 2% [76,121];

P-PPAR Ƴ synthetic derivative-treated group: receiving STZ + HFD as with positive control group, as well as the same treatment dose as reference pioglitazone (20 mg/kg/14 days, p.o) dissolved in Tween-80, 2%.

The two tested drugs were administrated for two consecutive weeks starting from the 24th day after the STZ injection. The doses depended on previous reference studies and a determined effective pilot study.

Synthesis and elucidation of P-PPAR Ƴ synthetic derivative;

The procedure is fully described in Supplementary material (S1);

STZ-induced diabetes mellitus type 2 model;

The procedure and mode of induction for diabetes mellites are fully described in Supplementary material (S2).

### 4.4. Isolation of Tissue and Preparation

After the rats received the last dose of treatment drugs, they were sacrificed by cervical dislocation under anesthesia. The aorta was gently freed of any adjacent tissues and lipids before undergoing midline thoracotomy. The exposed aorta tissue was divided into two portions: one was preserved at −80 °C until the assay time for endothelial tissue biomarker biochemical ELISA, qRt-PCR estimation for ET-1, E-selectin, VIP, and ICAM-1, and Western blotting estimation for p-Pi3k, p-AKT, p-PDK-1, p-mTOR, and qRT-PCR miR-126-5p; The second portion was preserved in formalin 10% isotonic solution for 48 h to adequate fixation before the histopathological examination, and an immunohistochemical assessment of eNOS expression was performed.

### 4.5. ELISA Determination for Specific Tissue Endothelial Biomarkers 

Previous cardioprotective studies indicated that increased endothelial content and leakage are valuable markers representing a pathological condition in the aortic strip tissues ET-1, E-selectin, VIP, and ICAM-1. These biomarkers were evaluated using ELISA chemical kits according to the instructions of the assay kit. According to the sandwich technique described previously, the assay depends on the colorimetric measurement of a microplate reader at 450 nm (Model Spectra Max Plus-384 Absorbance Microplate Reader, Molecular devices LLC (San Jose, CA, USA) to test the parameter levels [122].

### 4.6. Western Blot Analysis for PI3k/AKT/mTOR Signaling Pathway

Aortic cell lysis was performed using RIPA buffer (Beyotime Institute of Biotechnology) to evaluate p-AMPK, p-Pi3k, p-AKT, p-PDK, and p-mTOR expression; cell lysates were centrifuged at 10,000× *g* at 4 °C for 15 min. Using a bicinchoninic acid protein kit (Beyotime Institute of Biotechnology), protein quantification is as follows. Proteins were loaded on PVDF membranes after 10% SDS-PAGE separation; each lane contained 40 µg of protein. During this time, the membranes were soaked in a blocking solution of 5% nonfat milk in PBST (0.1% Tween-20) for 1 h at room temperature. Then, the samples were left overnight and incubated at 4 °C against the primary antibody. The membrane was then incubated against the secondary antibody at room temperature for an hour after being washed three times with PBST. Lastly, the protein bands were visualized using 5-bromo-4-chloro-3-indolyphosphate (BCIP)/nitro-blue tetrazolium (NBT). The quantification analysis of the detected bands was performed using Image-J/ NIH software and the BioRad microarray protein electrophoresis separation machine (Model 1658004, Sinorica International Patent and Trademark, Germantown, MD, USA). The assessment methodology is provided according to a previously described method [123]. 

### 4.7. Histopathological Study 

The aortic tissue strip slides were prepared for staining after being fixed for 24 h in a 10% formalin saline solution. Afterward, fixed tissues were transferred to hardening via paraffin blocks. Then, the aorta sections were cut and stained with standard hematoxylin and eosin (H&E) for histopathological investigations under a Nikon microscope at 400× magnification using Bancroft and Steven’s previously published method [120]. The slides were examined by a skilled pathologist.

### 4.8. Immunohistochemical Assay

The eNOS immunohistochemical investigation was performed using a previously described method [124]. The deparaffinized and rehydrated aortic tissues were washed with a buffer solution for 20 min. Then, the tissue was injected with an adequate digestive enzyme. Afterward, sections were exposed to 0.3% H_2_O_2_ for 10 min to decrease tissue endogenous peroxidase activity. At that point, the slides were incubated overnight at 4 °C with primary antibodies against eNOS. Following incubation, the slides were washed with buffer, reincubated with secondary antibody HRP for 10 min, and then washed with deionized water. Sections cleaned with deionized water were visualized by adding the DAB Quanto chromogen drop to 1 mL of DAB Quanto substrate. The slides were restained with hematoxylin. Finally, a professional observer used a light microscope (Leica microsystem, Wetzlar, Germany) to monitor the dehydration of slides in xylene and positive dye to identify the samples.

### 4.9. Quantitative Real-Time Polymerase Chain Reaction (qRT-PCR) for Determination of miR-126-5p Expression Levels

We used a Qiagen tissue extraction kit (Qiagen, Germantown, MD, USA) for aorta RNA extraction. Each stage was performed according to the manufacturer’s instructions. A NanoDrop^®^ ND-8000 UV–Vis spectrophotometer was used to determine the total RNA yield (NanoDrop Technologies, Wilmington, DE, USA). The full RNA isolation and identification are described in the Supplementary data (S3), along with the primer sequences.

### 4.10. Statistical Analysis

The mean and standard error of the mean (SEM) were used to depict the data in this study (eight participants). An ANOVA test followed by a Tukey–Kramer test on biochemical data was conducted using SPSS (version 19.0) computer software (SPSS Inc., Chicago, IL, USA). A *p*-value of 0.05 was considered statistically significant. Image J was used to measure the intensity of the bands on the Western blot (NIH, USA).

## 5. Conclusions

In conclusion, our results indicate that the newly designed partial PPAR Ƴ synthetic derivative, in addition to its anti-hypoglycemic potential, reduces the severity of vascular damage induced due to T2D through upregulating expression of microRNA126-5p, p-AKT/p-Pi3k/p-PDK 1/p-mTOR, and eNOS. In addition, the P-PPAR Ƴ synthetic derivative decreases endothelial inflammatory and vascular integrity parameters ET-1, ICAM-1, E-selectin, and VIP. The PPAR Ƴ synthetic derivative might become an alternative approach to improving diabetes vascular complications caused by metabolic syndrome insults mediated by the antioxidant, anti-inflammatory, and antiapoptotic signaling pathways. Further clinical trials are needed to confirm such findings clinically.

## Figures and Tables

**Table 1:** Serum fasting blood glucose levels after 14 days of treatment with oral PIO standard and P-PPARƳ synthetic derivative against STZ-induced diabetes mellitus. (Mean values, with standard errors (SEM) at *p* < 0.05); ^a^: significantly different from the control group; ^b^: significantly different from the STZ positive control group.

Groups	Fasting Blood Glucose mg/dL
Normal control	101.66 ± 4.73
Positive control	289.33 ± 12.90 ^a^
PIO	129.66 ± 8.08 ^b^
P-PPARƳ synthetic derivative	96.33 ± 8.14 ^b^

## Data Availability

Data is contained within the article and Supplementary Materials.

## Supplementary Materials

The following supporting information can be downloaded at: https://www.mdpi.com/article/10.3390/ph15101175/s1.
