# Supplementary material for: Partial Synthetic PPARƳ Derivative Ameliorates Aorta Injury in Experimental Diabetic Rats Mediated by Activation of miR-126-5p Pi3k/AKT/PDK 1/mTOR Expression
Source: Pharmaceuticals (Basel). 2022 Sep 22;15(10):1175. doi: 10.3390/ph15101175 (PMC9607084; doi:10.3390/ph15101175)
Supplement: Supplementary file 1 [file pharmaceuticals-15-01175-s001.zip › pharmaceuticals-1917195-supplementary.pdf]

Partial Synthetic PPARϒ Derivative Ameliorates Aorta Injury in Experimental Diabetic Rats Mediated by Activation of miR-126-5p Pi3k/AKT/PDK 1/mTOR Expression

Yasmin M. Ahmed [1], Raha Orfali [2,*], Nada S. Abdelwahab [3,4], Hossam M. Hassan [5], Mostafa E. Rateb [6] and Asmaa M. AboulMagd [3,*]

[1] Department of Pharmacology and Toxicology, Faculty of Pharmacy, Nahda University, Beni-Suef 62521, Egypt
[2] Department of Pharmacognosy, College of Pharmacy, King Saud University, P.O. Box 2457, Riyadh 11451, Saudi Arabia
[3] Department of Pharmaceutical Chemistry, Faculty of Pharmacy, Nahda University (NUB), Beni-Suef 62521, Egypt
[4] Department of Pharmaceutical Analytical Chemistry, Faculty of Pharmacy, Beni-Suef University, Beni-Suef 62521, Egypt
[5] Department of Pharmacognosy, Faculty of Pharmacy, Beni-Suef University, Beni-Suef 62521, Egypt
[6] School of Computing, Engineering & Physical Sciences, University of the West of Scotland, Paisley PA1 2BE, UK
* Correspondence: rorfali@ksu.edu.sa (R.O.); asmaa.aboulmaged@nub.edu.eg (A.M.A.)

Supplementary:

Supplementary Materials:

S1. General procedure for the synthesis of PPAR- γ synthetic derivative

To a solution of 4-oxo-4,5-dihydrothiazol-2-yl amino benzenesulfonamide (0.271gm, 0.001 mol) in glacial acetic acid (20 mL), sodium acetate (0.164gm, 0.002 mol) and vanillin (0.02 mmol) was added. The whole mixture was refluxed for 48 h, and TLC monitored the reaction till its completion of the reaction. The solid result was filtered and washed from ethanol several times. The residue was crystallized from acetic acid to afford the title compound.

Orange solid: yield, 82%; m.p. 263±2 ^{o}C; IR (KBr) ν_{max}:) 3561 (OH), 3360, 3271 (NH_2), 3201 (NH), 2862 (CH aliph.), 1678 (C=O) cm^{-1}, ^{1}H-NMR (DMSO-*d_6*) δ: 3.97 (s, 3H, OCH_3), 6.95 (d, *J*= 8.0 Hz, 1H, Ar-H), 7.35 (s, 1H, CH), 7.38 (m, 3H, Ar-H), 7.50 (s, 1H, OH), 7.55 (d, *J*= 8.0 Hz, 1H, Ar-H), 7.65 (d, *J*= 8.0 Hz, 1H, Ar-H), 7.84 (d, *J*= 8.0 Hz, 1H, Ar-H), 7.92 (s, 1H, NH), 7.99 (s, 2H, NH_2) ppm. ^{13}C-NMR (DMSO-*d_6*) δ: 57.0, 116.8, 117.0, 117.2, 122.0, 124.4, 127.0, 127.5, 129.1, 132.5, 133.2, 134.5, 136.4, 140.3, 144.9, 160.0, 160.8 ppm. **MS** (Mwt.: 405.05): m/z 405.44 (M+, 58.86%), 476.91 (47.73%), 352.81 (96.34%), 329.33 (61.38%), 284.90 (100%); Anal. Calcd. For $C_{17}H_{15}N_3O_5S_2$: C, 50.36; H, 3.73; N, 10.36. Found: C, 50.22; H, 3.94; N, 10.54 %.

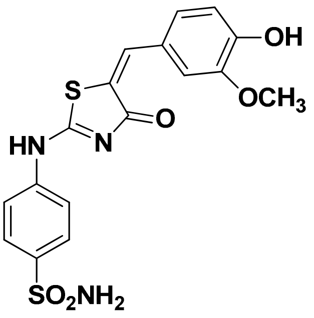

S2. Method for diabetes mellitus induction

Rats were fed a high-fat diet for three weeks before streptozotocin administration to induce insulin resistance. Rats were tested after an intraperitoneal injection of 45 mg/kg of freshly produced and dissolved citrate buffer streptozotocin for three weeks. To avert the animal's death, they allowed 20 percent glucose to be added to the drinking water for 48 hours following intraperitoneal streptozotocin injection. After 72 hours before streptozotocin administration, diabetic rats were defined as those with a blood glucose level greater than 300 mg/dL determined by a blood sample from rat tail (ACCU-CHEK®, Roche), USA) instruments. After diabetes induction and STZ-induced T2DM, the test agent was administered for 15 days. [23].

S3. Quantitative real time-Polymerase chain reaction (qRT-PCR) for determination of miR-126-5p expression levels

According to the kit provided protocol instructions, the extracted RNA was transcribed to cDNA using a high-capacity cDNA reverse transcription kit (ferments, USA). The isolated RNA was transcribed to cDNA according to the kit protocol (ferments, USA). The SYBR Green technique was used for qRT-PCR. 2 L cDNA, 10 L SYBR Green, 1 L of miR-126-5p F Primer: GCGCGCATTATTACTTTTGG and R Primer: TGGTGTCGTGGAGTCG, and 7 L nuclease-free water in a 20 L reaction mixture. Denaturation for 2 minutes at 95°C was followed by 45 cycles of 95°C for 10 seconds, 59°C for 20 seconds, and 72°C for 30 seconds. The expression of miR-126-5p were normalized using B-Actin, and the relative expression level of miR-126-5p was calculated using the 2-CT technique Primers were supplied by Sangon Biotech Co., Ltd.
